# Serial interferon-gamma release assays for latent tuberculosis in dialysis patients with end stage renal disease in a Korean population

**DOI:** 10.1186/s12879-015-1117-3

**Published:** 2015-09-21

**Authors:** Seung Heon Lee, Hee Jin Kim, Seok Ju Park, Tae Hee Kim, So Jeong Park, Sun Woo Kang, Yeong Hoon Kim, Dick Menzies

**Affiliations:** Division of Pulmonary, Sleep, and Critical Care Medicine, Department of Internal Medicine, Korea University Ansan Hospital, Korea University College of Medicine, Ansan, 425-707 Republic of Korea; Korean Institute of Tuberculosis, Cheongwongun, 363-954 Republic of Korea; Division of Nephrology, Department of Internal Medicine, Inje University Busan Paik Hospital, Inje University College of Medicine, Busan, 614-735 Republic of Korea; Respiratory Epidemiology and Clinical Research Unit, Montreal Chest Institute, McGill University, Montreal, QC H2X 2P4 Canada

**Keywords:** Interferon-gamma release assay, Latent tuberculosis, End stage renal disease, Dialysis, Reversion

## Abstract

**Background:**

Serial interferon-gamma-release-assay (IGRA) result can show variance due to within-subject variation and difference in host immune status, and may be affected by latent tuberculosis infection (LTBI) treatment. We aimed to know the changes in QFT-IT (QuantiFERON-TB Gold In-Tube) results measured at a 4 month interval in end stage renal disease patients and whether these changes were influenced by dialysis method or LTBI treatment.

**Methods:**

We prospectively performed serial QFT-IT tests at 4 month interval in 93 end stage renal disease (ESRD) patients on HD (hemodialysis) or PD (peritoneal dialysis). LTBI treatment was given to 18 of 39 patients with initial positive QFT-IT result. Agreement between the two results was estimated for all 93 patients and reversion rates were estimated among the 39 patients with initial positive QFT-IT.

**Results:**

Positive QFT-IT at the first and 2^nd^ tests were 41.9 and 34.4 %, respectively. The concordance rate between baseline QFT-IT and 2^nd^ QFT in 93 ESRD patients was excellent (90.3 %, kappa = 0.80, *p* < 0.001). Agreement between the first QFT-IT and 2^nd^ QFT-IT in HD (95.3 %, kappa = 0.91, *p* < 0.001) was higher than in PD patients (86.0 %, kappa = 0.69, *p* < 0.001). Among all ESRD patients, the odds of reversion of QFT-IT was not different in those who were, or were not treated for LTBI [odds ratio = 2.3 (0.5–11.4), *p* = 0.43].

**Conclusions:**

In a group of 93 dialyzed ESRD patients 8.6 % showed reversion of initial positive QFT to negative within 4 months. Reversion seemed not to be associated with LTBI treatment. Further study with larger numbers of patients is needed to investigate the variation of QFT-IT tests in dialyzed ESRD patients.

## Background

Tuberculosis (TB) remains an important global health issue, and is the single most significant infectious cause of morbidity and mortality in the world, with approximately 2 million deaths and around 9 million new cases each year [1–3]. LTBI diagnosis and treatment is a part of the strategy for TB elimination in countries with low or intermediate burden [4, 5].

There are several clinical conditions in which the risk for active TB is increased. ESRD patients as well as renal-transplant patients on immune suppressive agents who have LTBI have an increased risk for progressive active TB [6]. The annual incidence of active TB in South Korea where BCG vaccination is mandatory is intermediate (84/100,000) [7] and that of active TB in renal transplant patients is 0.5 %/year in South Korea [8]. Therefore, it would be better to treat LTBI in dialyzed ESRD patients before transplantation, because prophylactic TB drug like rifampicin can lead to drug interaction with immune suppression drug on kidney-transplantation recipients [9].

End stage renal disease (ESRD) is known to compromise cellular immune function, and patients on dialysis have been shown to have T cell dysfunction including abnormal IFN-gamma levels, considered to have a pivotal role in controlling latent TB infection [10, 11]. A high percentage of the ESRD patients show no skin reaction to tuberculin skin test (TST) as they are anergic [12, 13]. Interferon-gamma release assays (IGRAs) are useful tools for diagnosing LTBI, because they are more specific, particularly in BCG vaccinated populations [14], and may identify some additional patients with latent TB among kidney-transplantation (KT) recipients with a negative TST [15].

Therefore, IGRA may be useful in this patient population to detect TB infection, but the use of serial IGRA testing to detect new TB infection in these patients is controversial because of non-specific variation, conversions, and reversions. Lee *et al.* reported that QFT-IT test reversion occurred in 41.9 % of the subjects and IFN-gamma levels decreased in almost all subjects after LTBI treatment [16].

Although previous studies have proposed that conversions, reversions, and non-specific variations can occur with serial IGRA testing [17, 18], there are no studies about serial changes of IGRAs in patients with end stage renal disease who receive LTBI treatment. We prospectively evaluated QFT-IT tests on two occasions at a four month interval in dialyzed ESRD patients, some of whom received LTBI treatment, to estimate the variation of the QFT-IT on repeated testing, and the effect of dialysis type and LTBI therapy.

## Methods

### Study design

ESRD patients on Peritoneal dialysis (PD) or hemo-dialysis (HD) were enrolled in this study. QFT-IT test was performed initially for screening of LTBI, and the patients with positive QFT-IT started to receive 9 months of isoniazid treatment or 4 months of rifampicin treatment immediately. The QFT-IT test was repeated at 4 months interval for all the subjects including a sub-group who received Isoniazid for 9 months or rifampicin for 4 months- all of whom took > 80 % of doses. Chest radiographs were screened before LTBI treatment. Written informed consent was obtained from study participants, and the study was approved by the Institutional Review Board of the Inje University Busan Paik hospital.

### QuantiFERON-TB Gold In-Tube test

All participants were tested with the QFT-IT test per the manufacturer’s instructions. An IFN-gamma response to the ESAT-6/CFP-10/TB7.7 mixture ≥0.35 IU/mL above the nil control value (and ≥25 % of the nil control) was considered a positive result for the QFT-IT test. QFT-IT test reversion was defined as a change from a positive (≥0.35 IU/mL) to a negative (<0.35 IU/mL) result, and conversion as a change from a negative (<0.35 IU/mL) to a positive (≥0.35 IU/mL) result.

### Statistical analysis

All analyses were performed using SPSS software, version 12.0 (SPSS Inc., Chicago, IL, USA). Between-group comparisons were made with *t*-test and Mann–Whitney test for variables. Concordance between test results from first QFT-IT and 2^nd^ QFT-IT test was assessed using kappa coefficients. The *χ*^2^ tests was used to test for difference between reversion group and non-reversion group, and paired changes of interferon-gamma levels was analyzed by Wilcoxon signed rank test. All tests for significance were two-sided and statistical significance was established at *P* values < 0.05.

## Results

### Demographics of the participants

A total of 150 ESRD patients were screened with QFT-IT and chest radiographs. The QFT-IT test was repeated for the initially screened patients, but both baseline QFT-IT and 2^nd^ QFT-IT test results were available for 93 patients finally (Table 1). In other words, 2^nd^ QFT-IT test could not be performed for 57 ESRD patients (HD:22, PD:35), because 6 patients (HD:2, PD:4) were in inpatient care for associated disease, 13 patients (HD:6, PD:7) were transferred out to other hospital, and 38 patients (HD:14, PD:24) refused to participate in further study. Old TB lesion on chest radiograph was found on 35 (37.6 %) patients among them. Hemodialysis was used for 43 ESRD patients, and peritoneal dialysis was used for 50 ESRD patients. The mean age was 51.6 years (range, 23–74 years) and the male-to-female ratio was 43:50. A BCG scar was present in 62 patients (66.7 %). The positive rates of the 1^st^ QFT-IT at baseline testing and 2^nd^ QFT-GIT testing at the 4 month follow-up were 41.9 and 34.4 %, respectively. Isoniazid was prescribed for 10 patients and rifampicin was prescribed for 8 patients for the treatment of patients with LTBI showing positive QFT-IT results. There were no differences between the HD group and PD group in age, gender, BMI, positive rate of QFT-IT except the presence of BCG scar and the rate of LTBI treatment (Table 1).

**Table 1:** Clinical characteristics of the study population

Clinical characteristics	Total (*n* = 93)	HD (*n* = 43)	PD (*n* = 50)	*P-*value
Mean age, years (range)	51.6 (23–74)	52.8 (31–74)	50.6 (23–72)	0.41
Gender (Male : Female)	43:50	19:24	24:26	0.71
BMI, kg/m^2^, mean ± SD	23.0 ± 3.7	22.6 ± 3.1	23.3 ± 4.3	0.44
Cause for ESRD				
DM	34 (36.6)	19 (44.2)	15 (30.0)	0.23
HTN	44 (47.3)	19 (44.2)	25 (50.0)	0.73
^a^Others	15 (16.1)	5 (11.6)	10 (20.0)	0.66
Use of steroid	2 (2.2)	1 (2.3)	1 (2)	1.0
Previous TB treatment (%)	0 (0)	0 (0)	0 (0)	1.0
Presence of BCG scar (%)	62/93 (66.7)	19/43 (44.2)	43/50 (86.0)	0.001
Baseline QFT-IT + (%)	39/93 (41.9)	19/43 (44.2)	20/50 (40.0)	0.68
2nd QFT-IT + (%)	32/93 (34.4)	^b^19/43 (44.2)	13/50 (26.0)	0.07
Old TB lesion (%)	35/93 (37.6)	14/43 (32.6)	21/50 (42.0)	0.35
LTBI treatment among initial QFT-IT + (%)	18/39 (46.2)	5/19 (26.3)	13/20 (65.0)	0.02
Isoniazid: Rifampicin	10:8	3:2	7:6	1.0

^a^Others include autoimmune diseases, glomerulonephritis, polycystic kidney diseases, and unknown diseases

^b^One of 19 patients showed conversion from negative to positive result

*HD* Hemo-dialysis, *PD* peritoneal dialysis, *BMI* body mass index, *TB* tuberculosis, *LTBI* latent TB infection, *BCG* bacille Calmette-Guérin, *QFT-IT* QuantiFERON-TB Gold In-Tube, *SD* standard deviation

### Changes in QFT-IT result in ESRD patients between 0 and 4 months

The agreement between baseline QFT-IT and 2^nd^ QFT at 4 month interval in 93 ESRD patients was excellent (90.3 %, kappa = 0.80, *p* < 0.001) (Table 2). Agreement between baseline QFT-IT and 2^nd^ QFT-IT in HD (95.3 %, kappa = 0.91, *p* <0.001) was higher than in PD patients (86.0 %, kappa = 0.69, *p* < 0.001).

**Table 2:** 1^st^ QFT-IT and 2^nd^ QFT-IT test results in 93 ESRD patients

Dialysis	1^st^ QFT-IT positive (*n* = 39^a^)		1^st^ QFT-IT negative (*n* = 54^a^)		Total	Kappa	*P* value
	2^nd^ QFT-IT positive	2^nd^ QFT-IT negative	2^nd^ QFT-IT positive	2^nd^ QFT-IT negative	(N)	(%)	
HD	18/43 (41.9 %)	1/43 (2.3 %)	1/43 (2.3 %)	23/43 (53.5 %)	43	0.91 (95.3 %)	<0.001^*^
PD	13/50 (26.0 %)	7/50 (14.0 %)	0/50 (0 %)	30/50 (60.0 %)	50	0.69 (86.0 %)	<0.001^*^
Total	31/93 (33.3 %)	8/93 (8.6 %)	1/93 (1.1 %)	53/93 (57.0 %)	93	0.80 (90.3 %)	<0.001^*^

^a^Among 93 ESRD patients, 39 patients showed QFT-IT positive results and 54 patients showed QFT-IT negative results on baseline QFT-IT tests, respectively

^*^Difference for concordance between 1^st^ QFT-IT and 2^nd^ QFT-IT

*QFT-IT* QuantiFERON-TB Gold In-Tube, *PD* peritoneal dialysis, *HD* hemodialysis

### Changes in QFT-IT among ESRD patients with 1^st^ QFT-positive results

Among 39 patients who showed positive QFT-IT result at first testing, QFT-IT test reversion occurred in 8 (20.5 %) patients, of whom seven (87.5 %) were on PD, and one patient (12.5 %) was on HD [odds ratio = 9.7 (1.1–88.7), *p* = 0.04] (Table 3). However, conversion rate was not different between them (0 vs. 2.3 %, *p* > 0.05). The median actual interferon-gamma levels of QFT-IT in HD group and PD group at baseline were; 1.55 (IQR 0.7–3.7) and 5.03 (IQR 0.9–15.4) respectively. The median values after 4 months were: HD = 0.87 (IQR 0.6–3.4), and PD = 1.31 (IQR 0.2–4.5). The change in median values of QFT-IT between baseline and 4 months was significantly different with HD = 8 % decrease, and PD = 76 % (*p <* 0.001)

**Table 3:** Reversion and non-reversion of QFT-IT among ESRD patients with 1^st^ QFT-IT positive result

ESRD patients (*N* = 39)		Reversion	Non-reversion	OR (95 % CI)	*P* value
Dialysis method					
	PD	7 (35.0 %)	13 (65.0 %)	9.7 (1.1–88.7)	0.04
	HD	1 (5.3 %)	18 (94.7 %)	1 (Ref.)	
Treatment					
	LTBI treatment	5 (27.8 %)	13 (72.2 %)	2.3 (0.5–11.4)	0.43
	No LTBI Treatment	3 (14.3 %)	18 (85.7 %)	1 (Ref.)	
Treatment drug					
	Rifampin	3 (37.5 %)	5 (62.5 %)	2.4 (0.3–19.8)	0.61
	INH	2 (20.0 %)	8 (80.0 %)	1 (Ref.)	

*OR* odds ratio, *PD* peritoneal dialysis, *HD* hemodialysis, *LTBI* latent tuberculosis infection, *RFP* rifampicin, *INH* isoniazid

**Fig. 1:**
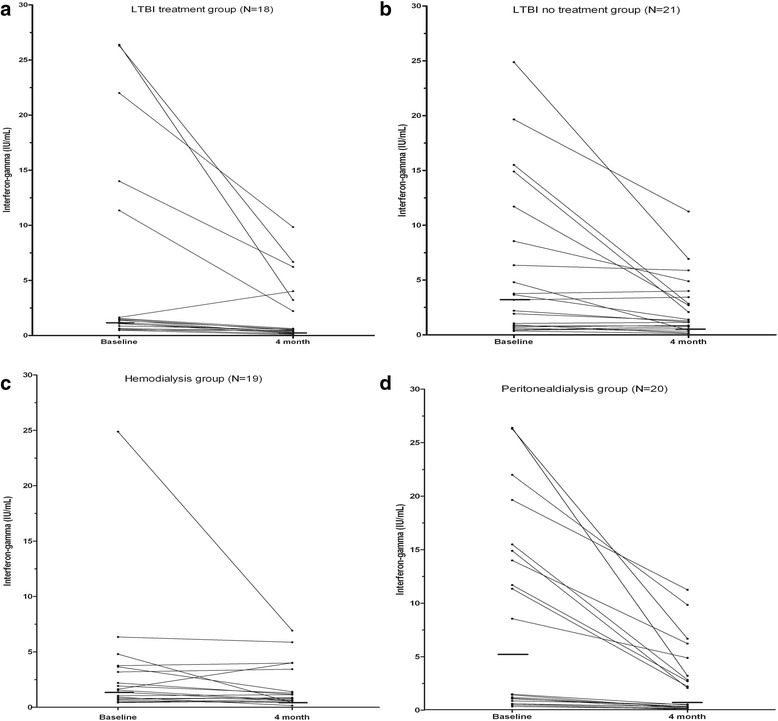
Changes of interferon-gamma levels over 4 months in those with positive QFT-IT at baseline. Paired changes of interferon-gamma levels in QFT-IT tests after 4 months in the LTBI treatment group (**a**) and in the LTBI no treatment group (**b**), as well as in the hemodialysis group (**c**) and in the peritoneal dialysis group (**d**). The horizontal bold lines at pre- and post-treatment values represent the median interferon-gamma values (**a** median 1.40 with IQR 0.8–12.0 and median 0.51 with IQR 0.3–3.4, **b** median 3.19 with IQR 0.8–10.1 and median 1.29 with IQR 0.7–3.7, **c** median 1.55 with IQR 0.7–3.7 and median 0.87 with IQR 0.6–3.4, **d** median 5.03 with IQR 0.9–15.4 and median 1.31 with IQR 0.2–4.5). QFT-IT: QuantiFERON-TB Gold In-Tube; IQR: interquartile range

Among the 39 with baseline positive IGRA tests, there was no significant difference in the percent decline in interferon-gamma levels of QFT-IT tests over 4 months among the 18 who received LTBI treatment group and the 21 who were not treated (Decrease of 64 % among treated vs. 43 % among untreated, *p* > 0.05). However, as seen in Fig. 1, median values decreased significantly in both groups over the 4 month interval. Reversion odds of the QFT-IT test in the groups who received, or did not receive LTBI treatment were not significant [odds of reversion = 2.3 (0.5–11.4)]. Furthermore, there were no difference in reversion odds of the serial QFT-IT test between those treated with RIF or INH [odds ratio = 2.4 (0.3–19.8)].

## Discussion

Within-subject variability of QFT-IT at 4 month interval in dialyzed ESRD patients was minimal. Reversion of QFT-IT after a 4 month interval was not related to LTBI treatment but was more frequent in PD patients than in HD patients.

QFT-IT in our study was checked in ESRD patients; the concordance rate in all ESRD patients was relatively high (90.3 %, kappa = 0.80), compared with previous study in which concordance rate at 1 year interval without LTBI treatment in ESRD patients was 70.4 % with T-SPOT-TB and 73.5 % with QFT-IT [19]. Furthermore, reversion rates after LTBI treatment in our study were relatively low (27.8 %, 5/18), compared with previous study in which IGRA reversion rates were 41.9 % with 4 months of rifampicin treatment [16]. Also, in case of conversion, only 1.9 % (1/54) HD patient showed conversion of QFT-IT in our study, which was relatively lower than 12.1 % in patients who was taking the immune-suppressive drugs at 8.5 month interval [20] and 6 % in ordinary patients with infectious disease at 3 month interval [21]. Compared with these studies, our result for ESRD patients with 4 month follow up showed low variance of IFN-gamma level. The reason why within-subject variability was low seems to be due to immune dysfunction of ESRD patients related to uremia and dialysis [11] as well as due to short term interval of QFT-IT follow up.

In our study, there is possibility that the LTBI treatment reduced the IFN-gamma level because 2^nd^ IFN-gamma level decreased from baseline level more frequently in LTBI treatment group (17/18, 94.4 %) than in LTBI no treatment group (14/21, 66.7 %) (*p* = 0.049). However, the 2^nd^ IFN-gamma level did not sufficiently decrease to reach to the defined cut-off level 0.35 IU/mL, and there were no difference in degree of decrease between two group (median value of percent decrease, 64.0 VS. 42.8 %, *p* > 0.05). Therefore, the threshold for IFN-gamma cut-off point must be considered to be re-defined which is optimal for distinguishing true reversion and non-specific variation.

The main reasons for the predominant reversion of QFT-IT in PD patients compared with HD patients may be the differences in immune activity and serum sampling time as well as in LTBI treatment rate (65.0 VS. 26.3 %, *P* = 0.02) between PD patients and HD patients. As seen in Fig. 1c and d, the median IFN-gamma levels on two time point were higher in PD patents than in HD patients, and the percent decrease in IFN-gamma level from baseline QFT-IT to sequential 4 month QFT-IT was significantly higher in PD patients than in HD patients (median value of percent decrease, 75.5 VS. 8.0 %, *p <* 0.001). This result could be occurred by accident, but T-SPOT TB which was reported to be equivalent to or inferior to QFT-IT in sensitivity [22] can be tested for reproducibility in PD patients to quantify the degree of decrease as another method. The explainable hypothesis for the difference of immune activity between HD and PD patients is that lymphopenia, decreased absolute counts of lymphocyte, and decreased production of interferon-gamma levels are more apparent in HD patients than in PD patients [23]. Circulating immune cells in PD patients may be more easily exhausted by chronic uremic activation [24]. Also, the timing in collecting the blood sample for QFT-IT could influence the variation of QFT-IT result of PD, because it was reported that the IFN-gamma level of post-dialysis could be reduced compared with that of pre-dialysis [25]. In our study, blood sampling was performed equally before dialysis in HD patients, but in PD patients, not equally before or after dialysis.

This study had some limitations. Firstly, the number of participants was small and the tuberculin skin tests were not performed in most of patients due to difficulty in follow up schedule for interpretation of TST results. Secondly, the timing for blood sampling in dialysis patients could not be equally conditioned for all subjects. Lastly, because the follow up interval is short, the serial extended follow up of QFT-IT at 9 months after LTBI treatment for more than 4 months is needed for confirming the variation of within-subject variability.

## Conclusions

The concordance rate of QFT-IT for 4 month interval in dialyzed ESRD patients in a Korean population was excellent, and reversion seemed not to be associated with LTBI treatment. Further studies with extended follow up as well as with larger numbers of patients are needed to investigate the changes of QFT-IT including the effect of LTBI treatment in dialyzed ESRD patients.
